# Development of Primer Sets for Loop-Mediated Isothermal Amplification that Enables Rapid and Specific Detection of *Streptococcus dysgalactiae*, *Streptococcus uberis* and *Streptococcus agalactiae*

**DOI:** 10.3390/ijerph120605735

**Published:** 2015-05-26

**Authors:** Deguo Wang, Yanhong Liu

**Affiliations:** 1 Henan Postdoctoral Research Base, Food and Bioengineering College, Xuchang University, Xuchang 461000, China; E-Mail: wangdg666@aliyun.com; 2 Molecular Characterization of Foodborne Pathogens Research Unit, Eastern Regional Research Center, Agricultural Research Service, U.S. Department of Agriculture, 600 East Mermaid Lane, Wyndmoor, PA 19038, USA

**Keywords:** loop-mediated isothermal amplification (LAMP), 16S rRNA gene, 16S-23S rRNA intergenic spacer, *Streptococcus dysgalactiae*, *Streptococcus uberis*, *Streptococcus agalactiae*

## Abstract

*Streptococcus dysgalactiae*, *Streptococcus uberis* and *Streptococcus agalactiae* are the three main pathogens causing bovine mastitis, with great losses to the dairy industry. Rapid and specific loop-mediated isothermal amplification methods (LAMP) for identification and differentiation of these three pathogens are not available. With the 16S rRNA gene and 16S-23S rRNA intergenic spacers as targets, four sets of LAMP primers were designed for identification and differentiation of *S. dysgalactiae*, *S. uberis* and *S. agalactiae*. The detection limit of all four LAMP primer sets were 0.1 pg DNA template per reaction, the LAMP method with 16S rRNA gene and 16S-23S rRNA intergenic spacers as the targets can differentiate the three pathogens, which is potentially useful in epidemiological studies.

## 1. Introduction

Bovine mastitis (BM) is a persistent and inflammatory reaction of the udder tissue, usually due to microbial infection [1]. The potentially fatal mammary gland infection is the most common disease in dairy cattle, which is also the most costly to the dairy industry [2]. According to the mode of transmission, mastitis can be classified as contagious mastitis (mainly caused by *Staphylococcus aureus* and *Streptococcus agalactiae*) or environmental mastitis (*Streptococcus dysgalactiae*, *Streptococcus uberis*, *Streptococcus* *parauberis* and *Escherichia coli*) [3]. Bovine mastitis can also be divided into clinical mastitis and sub-clinical mastitis, according to the clinical symptoms.

Clinical mastitis can be identified by abnormalities in the udder such as swelling, heat or redness as well as by milk abnormalities such as a watery appearance, flakes, or clots. In contrast, there is no visible sign of infection when cow is infected with subclinical mastitis, and a method of rapidly identifying sub-clinical mastitis is needed for the control of infection in the herd [4,5].

Polymerase chain reaction (PCR) assays had been developed to identify pathogens in bovine mastitis [6], this molecular method used for analyzing pure microbial cultures is very effective [7], but limited by PCR inhibitors present in biological and food samples, which results in reduction of the detection sensitivity or production of false-negative results [8,9]. Therefore, rapid, sensitive, and cost-effective methods are needed for identification and differentiation of bovine mastitis pathogens.

Loop-mediated isothermal amplification (LAMP) developed by Notomi *et al.* in 2000 [10], can rapidly amplify nucleic acids by utilizing a DNA polymerase enzyme with high strand displacement activity and two pairs of primers recognizing six independent sequences of a target gene under isothermal conditions with great specificity and sensitivity. Moreover, in 2002 Nagamine *et al.* advanced this method by introducing forward loop primers that accelerated the LAMP reaction [11]. Due to the cost effectiveness and sensitivity of LAMP, there has been significant interest in application of this method toward basic research in medicine and environmental testing, as well as point-of-care testing and diagnosis of infectious diseases in clinical settings [12]. LAMP has also been widely applied in pathogen detection including *Escherichia coli* O157:H7 [13], *Actinobacillus actinomycetemcomitans* [14], *Mycobacterium tuberculosis* [15], *S. pneumonia* [16], and *Listeria monocytogenes* [17]. More recently, LAMP has been used successfully to detect the bovine mastitis pathogens *Staphylococcus aureus* [18,19] and *S. agalactiae* [20].

In this paper, a LAMP method was developed for the identification and differentiation of *S. dysgalactiae*, *S. uberis* and *S. agalactiae.* Four sets of LAMP primers were designed targeting the 16S rRNA gene and 16S-23S rRNA intergenic spacers, and tested for the sensitivity and specificity in LAMP reactions.

## 2. Experimental Section

### 2.1. Bacterial Strains, Culture Conditions and Genomic DNA Isolation

Twenty five bacterial strains including *S. dysgalactiae* subsp. *equisimilis* strain ATCC 9542, *S. uberis* ATCC 700407 and *S. agalactiae* ATCC 27956 used in this study are listed in Table 1. *Listeria* strains were cultured overnight at 37 °C in Difco^TM^ Buffered Listeria Enrichment Broth Base (Becton, Dickinson and Company, Franklin Lakes, NJ, USA) while other strains were cultured overnight at 37 °C in Luria-Bertani (LB) broth. Genomic DNA from the overnight cultures was extracted using DNeasy® Blood & Tissue Kit (Qiagen, Inc., Valencia, CA, USA) according to the manufacturer’s instructions.

**Table 1:** Bacterial strains used in this study and specificity of four LAMP primer sets.

Bacterial Strains	Primer Set I	Primer Set II	Primer Set III	Primer Set IV
*Streptococcus dysgalactiae* ATCC 9542	4 **^a^**/4 **^b^**	4/4	0/4	0/4
*Streptococcus uberis* ATCC 700407	4/4	0/4	4/4	0/4
*Streptococcus agalactiae* ATCC 27956	4/4	0/4	0/4	4/4
*Staphylococcus aureus* ATCC 25923	0/4	0/4	0/4	0/4
*Listeria monocytogenes* J2-020 (1/2a)	0/4	0/4	0/4	0/4
*Listeria monocytogenes* J2-064 (1/2b)	0/4	0/4	0/4	0/4
*Listeria monocytogenes* J1-169 (3b)	0/4	0/4	0/4	0/4
*Listeria monocytogenes* J1-049 (3c)	0/4	0/4	0/4	0/4
*Listeria monocytogenes* M1-004 (N/A)	0/4	0/4	0/4	0/4
*Listeria monocytogenes* J1-094 (1/2c)	0/4	0/4	0/4	0/4
*Listeria monocytogenes* C1-115 (3a)	0/4	0/4	0/4	0/4
*Listeria monocytogenes* J1-031 (4a)	0/4	0/4	0/4	0/4
*Listeria monocytogenes* W1-110 (4c)	0/4	0/4	0/4	0/4
*Listeria monocytogenes* ATCC19115 (4b)	0/4	0/4	0/4	0/4
*Listeria innocua* ATCC51742	0/4	0/4	0/4	0/4
*Escherichia coli* O157:H7 933	0/4	0/4	0/4	0/4
*Listeria invanovii* ATCC49954	0/4	0/4	0/4	0/4
*Salmonella typhimuriam*	0/4	0/4	0/4	0/4
*Escherichia coli* O111:H8	0/4	0/4	0/4	0/4
*Salmonella enterica* serotype Newport	0/4	0/4	0/4	0/4
*Escherichia coli* O26:H11	0/4	0/4	0/4	0/4
*Escherichia coli* O121:H19	0/4	0/4	0/4	0/4
*Escherichia coli* O103:H2	0/4	0/4	0/4	0/4
*Escherichia coli* O145:H2	0/4	0/4	0/4	0/4
*Escherichia coli* O45:H12	0/4	0/4	0/4	0/4

**^a^** Numbers of LAMP positive; **^b^** Numbers of total experiments carried out.

### 2.2. Primer Design

Sequences targeting the specific 16S rRNA gene (GenBank Locus: AP011114.1) of *Streptococcus* spp., the 16S-23S rRNA intergenic spacer (GenBank Locus: AY351330.1) of *S. dysgalactiae* subsp. equisimilis strain ATCC 9542, the 16S-23S rRNA intergenic spacer (GenBank Locus: AY347567.1) of *S. uberis* ATCC 700407, and the 16S-23S rRNA intergenic spacer (GenBank Locus: DQ204552.1) of *S. agalactiae* ATCC 27956 were used to design primers. Four sets of LAMP primers designed using PrimerExplorer 4 and Oligo 7 are listed in Table 2 [21].

**Table 2:** Primers for identification and differentiation of *S. dysgalactiae*, *S. uberis* and *S. agalactiae* with LAMP method.

Target	Primer	Sequence (5’–3’)
16S rRNA gene of *Streptococcus* spp. (Primer set I)	FIP	CGGCACTAAGCCCCGGAAAGTTTTGTAGTCCACGCCGTAAACG
	BIP	CTGGGGAGTACGACCGCAAGTTTTCATGCTCCACCGCTTGTG
	F3	GTGGGGAGCAAACAGGATT
	B3	CCTGGTAAGGTTCTTCGCG
	LF	GGCCTAACACCTAGCACTCAT
	LB	GTTGAAACTCAAAGGAATTGACGG
16S-23S rRNA intergenic spacer of *S. dysgalactiae* (Primer set II)	FIP	TAATGGAGCCTAGCGGGATCTTTTTTAGCTCAGCTGGGAGAG
	BIP	TGTCCATTGAAAATTGAATATCTTTTTTCTTGTTACTATTCGTACAATCA
	F3	GTTTTGAGAGGTCTTGTGG
	B3	TTCACAGCGTTTTCGGTT
	LF	TGCGTGCAAAGCAGGCG
16S-23S rRNA intergenic spacer of *S. uberis* (Primer set III)	FIP	CTCTCCCAGCTGAGCTAAGGTTTTATTTAGTTTTGAGAGGTCTT
	BIP	ATCCCGCTAGGCTCCATAGGTTTTCAATGGACTATACTAAGATACAATG
	F3	ACACGTTGGTTAAGTCTT
	B3	TTTCATGATCGTGGAATT
	LF	CCCCACAGTTTGTCTCTG
	LB	ATACAGTTCAACTGACCT
16S-23S rRNA intergenic spacer of *S.* agalactiae (Primer set IV)	FIP	CAATGGAGCCTAGCGGGATCTTTTCTTAGCTCAGCTGGGAGA
	BIP	ATATCAAATTCCACGATCTAGAAATTTTTTTCACAGCGTTTTCGGTT
	F3	AGTTTTGAGAGGTCTTGTG
	B3	GTTTCTTTAAAACTAGAAAACTCA
	LF	CTGACCTCCTGCGTGCAAAGC

### 2.3. Sensitivity of the LAMP Method

LAMP was performed in a 25 μL reaction mixture containing 0.8 mM each of FIP and BIP, 0.2 mM each of F3 and B3, 0.4 mM each of LF and LB, 1.0 mM dNTPs, 20 mM Tris-HCl (pH 8.8), 10 mM KCl, 10 mM (NH_4_)_2_SO_4_, 6 mM MgSO_4_, 0.1% Triton X-100, 7.5% DMSO [22], 1× EvaGreen, 1× Rox, serial dilutions of related DNA templates (*S. dysgalactiae* subsp. equisimilis strain ATCC 9542, *S. uberis* ATCC 700407 or *S. agalactiae* ATCC 27956) ranging from 0.01–1000 pg, and 3.2 U Bst 2.0 WarmStart DNA polymerase (New England Biolabs, Beverly, MA, USA) [23]. The reaction mixtures were heated at 57°C for 60 min in a StepOne^TM^ System and the detection limit of conventional LAMP was determined. Negative control (no template DNA, only Tris-EDTA buffer) was included in every reaction batch.

### 2.4. Specificity of the LAMP Method

Twenty five bacterial strains including *S. dysgalactiae* subsp. *equisimilis* strain ATCC 9542, *S. uberis* ATCC 700407 and *S. agalactiae* ATCC 27956 (Table 1) were used to test the specificity of the LAMP method. 100 pg of genomic DNA were used for each reaction.

## 3. Results and Discussion

### 3.1. Detection Limits of the LAMP Method

The LAMP mixtures with the designed primers were used to detect a serial dilution of *S. dysgalactiae* subsp. equisimilis strain ATCC 9542, *S. uberis* ATCC 700407 or *S. agalactiae* ATCC 27956 DNA template, which were heated at 57°C for 60 min. As shown in Table 3, the detection limit of all four LAMP primer sets were 0.1 pg DNA template per reaction with no detectable false-positive response.

**Table 3:** Detection limits of four LAMP primer sets for identification and differentiation of *Streptococcus dysgalactiae*, *Streptococcus uberis* and *Streptococcus agalactiae*.

Bacterial strains	Primer Set I	Primer Set II	Primer Set III	Primer Set IV
*S. dysgalactiae*	0.1 pg (4 **^a^**/4 **^b^**)	0.1 pg (4/4)	**–**	**–**
*S. uberis*	0.1 pg (4/4)	**–**	0.1 pg (4/4)	**–**
*S. agalactiae*	0.1 pg (4/4)	**–**	**–**	0.1 pg (4/4)

Notes: Primer Set I: Method with primers targeted at 16S rRNA gene of *Streptococcus* spp; Primer Set II: Method with primers targeted at 16S-23S rRNA intergenic spacers of *S. dysgalactiae*; Primer Set III: Method with primers targeted at 16S-23S rRNA intergenic spacers of *S. uberis*; Primer Set IV: Method with primers targeted at 16S-23S rRNA intergenic spacers of *S. agalactiae*. Non-specific amplification of negative controls (false-positives) were not detected for each primer set; **^a^** Numbers of LAMP positive; **^b^** Numbers of total experiments carried out.

### 3.2. Specificity of the LAMP Method

Primers targeting the 16S rRNA gene of *Streptococcus* spp. were used to test the specificity of the LAMP method. In addition, *S. dysgalactiae* subsp. equisimilis strain ATCC 9542, *S. uberis* ATCC 700407 and *S. agalactiae* ATCC 27956 were also included in the LAMP assay. As shown in Table 1, these three strains were successfully detected. 22 non*-Streptococcus* strains were also included in the LAMP assays, all four repeated reactions of any non-*Streptococcus* strain were negative.

The LAMP method with primer sets targeting the 16S-23S rRNA intergenic spacers of *S. dysgalactiae*, *S. uberis* and *S. agalactiae* were also tested with25 strains (Table 1). *S. dysgalactiae* subsp. equisimilis strain ATCC 9542, *S. uberis* ATCC 700407 and *S. agalactiae* ATCC 27956 were successfully detected, respectively, while all other reactions were negative (Table 1).

Therefore, the four LAMP primer sets presented here can identify and differentiate *S. dysgalactiae*, *S. uberis* and *S. agalactiae* with high specificity.

## 4. Conclusions

Phenotypic characteristics cannot be used to identify sub-clinical mastitis, and bacterial culturing methods are complex and labor intensive, therefore, genotypic methods are generally used for bacterial identification for sub-clinical mastitis. Molecular methods based on the 16S rRNA gene sequences are robust, reproducible, and accurate [24]. In this study, a set of LAMP primers (primer set I) targeting the 16S rRNA gene of *Streptococcus* spp. was designed for loop-mediated isothermal amplification (LAMP), the LAMP method can be successfully used to identify of *Streptococcus* spp. at the genus level.

16S-23S rRNA intergenic spacers have been used to differentiate probiotic lactic acid bacteria [25], acetic acid bacteria [26], and *Vibrio* species [27]. A previous study showed that staphylococci and streptococci that cause bovine mastitis can be identified via 16S-23S rRNA intergenic spacers [28]. Therefore, three sets of LAMP primers targeting the 16S-23S rRNA intergenic spacers of *S. dysgalactiae*, *S. uberis* and *S. agalactiae* were designed, respectively. The corresponding three LAMP primer sets can favorably differentiate *S. dysgalactiae*, *S. uberis* and *S. agalactiae* at species levels.

The LAMP method with four sets of primers developed in this study are rapid, sensitive and specific, which may be helpful in field studies of diagnosis and effective treatment, antibiotic selection, and control of mastitis.
